# Anxiety, Social Support, Family Resilience, and Quality of Life Among Women Undergoing In Vitro Fertilization: A Cross-Sectional Mediation Analysis

**DOI:** 10.3390/healthcare14121632

**Published:** 2026-06-09

**Authors:** Jie Bai, Jinxia Zheng, Ke Wang, Yueyan Dong, Ying Liu, Hui Jiang

**Affiliations:** 1 Shanghai Key Laboratory of Maternal Fetal Medicine, Shanghai Institute of Maternal-Fetal Medicine and Gynecologic Oncology, Shanghai First Maternity and Infant Hospital, School of Medicine, Tongji University, Shanghai 200092, China; baijie1@51mch.com (J.B.); zjxxdx@51mch.com (J.Z.); wangke@51mch.com (K.W.); dongyueyan@51mch.com (Y.D.); 2 Clinical Research Unit, Shanghai Key Laboratory of Maternal Fetal Medicine, Shanghai Institute of Maternal-Fetal Medicine and Gynecologic Oncology, Shanghai First Maternity and Infant Hospital, School of Medicine, Tongji University, Shanghai 200092, China

**Keywords:** anxiety, quality of life, in vitro fertilization, social support, family resilience

## Abstract

**Background:** Women undergoing in vitro fertilization (IVF) often experience poor well-being. Social support and family resilience are considered protective factors, but their combined roles in the anxiety–fertility-related quality of life (FertiQoL) relationship remain unclear. **Design:** A cross-sectional study was conducted with women undergoing IVF at the reproductive medicine department of a tertiary hospital in Shanghai, China, between March and December 2024. **Methods:** Participants completed validated measures of anxiety, social support, family resilience, and QoL. Associations and indirect effects were examined using mediation analysis, and sensitivity analyses were conducted. **Results:** A total of 419 women participated in the study (response rate = 98.6%; mean age = 33.3 ± 4.7 years). Anxiety was negatively associated with social support, family resilience, and FertiQoL (all *p* < 0.001). The total indirect association between anxiety and FertiQoL involving social support and family resilience was statistically significant (B = −0.15, *p* = 0.03). However, the specific indirect effects, including the hypothesized sequential pathway through social support and family resilience, were not statistically significant. The total association between anxiety and FertiQoL was significant (β = −0.32, *p* < 0.001). **Conclusions:** Anxiety was associated with poorer FertiQoL among women undergoing IVF. Although the findings suggest an overall indirect association involving social support and family resilience, the hypothesized sequential mediation pathway was not supported. Therefore, the results should be interpreted cautiously, and longitudinal studies are needed to clarify the temporal and causal relationships among these psychosocial factors.

## 1. Introduction

Infertility is a prevalent condition affecting millions of couples of reproductive ages. The WHO reported that approximately one in six individuals worldwide now experiences infertility [1]. A prospective cohort study in Shanghai, China, reported an overall infertility incidence of 17%, with rates of 19% for couples with a “first-child intention” and 4% for those with a “second-child intention” [2]. Beyond its physical and economic toll, infertility profoundly impacts the mental health and well-being of couples [3]. This psychological burden persists from diagnosis through the end of treatment, particularly following unsuccessful cycles [4]. For Chinese couples seeking fertility treatment, the strong sociocultural emphasis on childbearing within marital and family contexts renders them particularly vulnerable to psychological distress [5]. A growing body of research across the world, including in countries such as China [6], India [7], and Italy [8], suggests that demographic factors, including income, education, and geographic location, significantly influence both the prevalence and severity of psychological distress among infertile individuals. These demographic factors likely shape the acceptability, accessibility, and utilization of fertility treatments, as well as the distress associated with infertility.

Anxiety during fertility treatments like in vitro fertilization (IVF) has been shown to impair emotional well-being, cognitive function, and overall quality of life (QoL) [9]. Infertile couples in China, especially women, frequently report elevated levels of anxiety and depression [6]. A systematic review found that, compared with women without infertility, women experiencing infertility exhibit a higher prevalence of mental health disorders, including anxiety and depression [10]. Understanding the mechanisms linking anxiety to QoL is therefore critical for developing psychosocial interventions to support women undergoing fertility treatment.

Fertility clinicians must also consider the sociocultural forces shaping patients’ psychological experiences. In the Chinese context, cultural attitudes toward childbearing influence engagement with fertility care and levels of distress [5]. The pressure to conceive is often intertwined with family expectations and community perceptions, making infertility not only a medical issue but also a deeply social and relational one. Consequently, infertility-related anxiety must be understood within this cultural framework of family obligations, social support, and shared expectations.

The interplay between individual psychological states and family or social resources is particularly relevant for shaping health outcomes. According to social support theory [11], support from family, friends, and significant others provides emotional, informational, and practical resources that may be associated with better coping and psychological adjustment [12]. In the context of infertility, social support has been linked to lower anxiety and better emotional adaptation [13]. Family resilience theory further emphasizes adaptive family processes, such as effective communication, flexibility, and problem-solving, which may be associated with resilience among family members and psychological well-being [14]. Integrating these frameworks, the present study proposed a theoretically informed psychosocial pathway in which anxiety is associated with social support, family resilience, and QoL. This proposed sequence reflects a conceptual progression from individual-level psychological distress to interpersonal-level resources and family-level adaptive capacity. Social support was specified before family resilience because supportive relationships may represent a proximal interpersonal resource associated with broader family adaptive processes. However, this ordering should be interpreted as theoretical rather than causal, and alternative or reciprocal relationships among anxiety, social support, family resilience, and QoL are also plausible.

Both theory and empirical evidence inform the rationale for this model. Social support theory suggests that supportive relationships may provide emotional, informational, and practical resources associated with better psychological adjustment [15]. In contrast, family resilience theory emphasizes adaptive family processes, such as communication, flexibility, and problem-solving, that may be related to well-being under stress [16]. Empirically, social support has been associated with psychological and family resilience, both of which have been linked to anxiety and depression among women undergoing fertility treatment [17]. For example, a study of 492 infertile Chinese women reported that psychological distress was positively associated with infertility-related stress but negatively associated with family resilience. Simple slopes analysis further revealed that infertility-related stress had a weaker positive association with psychological distress among women with higher family resilience compared with those with lower family resilience [18].

Although social support and family resilience have been associated with psychological adjustment among women undergoing IVF, few studies have examined whether these psychosocial resources are involved in indirect associations between anxiety and QoL. Understanding this interaction is important because social support may serve as a proximal resource that facilitates the development of broader family resilience, which in turn could buffer the adverse effects of anxiety.

## 2. Study Aims and Hypotheses

The current study aimed to: (1) examine the association between anxiety and FertiQoL among women undergoing IVF; and (2) explore whether social support and family resilience were involved in theory-guided indirect associations between anxiety and FertiQoL.

### Integrated Conceptual Model

**Figure 1:**
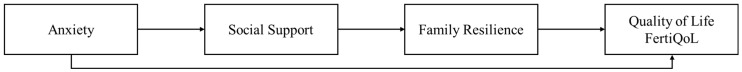
Conceptual model.

Integrating social support and family resilience theories, we proposed a theoretically informed psychosocial pathway in which anxiety is associated with social support, family resilience, and FertiQoL (Figure 1). The proposed model was specified as:

Anxiety (X) → Social Support (M_1_) → Family Resilience (M_2_) → Quality of Life (Y)

We hypothesized an indirect association between anxiety and quality of life through the sequential pathway of social support and family resilience. This proposed pathway reflects the possibility that individual distress, interpersonal resources, and family-system resources may be interconnected in relation to FertiQoL.

## 3. Materials and Methods

### 3.1. Study Design

A single-center, cross-sectional study was conducted to examine theory-guided indirect associations among anxiety, social support, family resilience, and FertiQoL.

### 3.2. Setting

This study was conducted at the reproductive medicine department of a tertiary hospital in Shanghai, China.

### 3.3. Study Size

Methodological recommendations for sequential mediation analysis guided sample size determination. Simulation studies suggest that for models with two sequential mediators, sample sizes of 300–500 provide adequate power (≥0.80) to detect medium indirect effects at an α level of 0.05 [19]. Based on these recommendations and accounting for potential attrition, we targeted a sample size of 350 participants. This sample size exceeds the minimum requirements for detecting individual path coefficients while providing sufficient power for testing the sequential indirect effect.

### 3.4. Participants and Recruitment

Participants were women currently undergoing IVF treatment, including ovarian stimulation, embryo transfer, or the two-week wait period, who were able to read and understand the local language and provide informed consent. Women were excluded if they had cognitive impairments affecting questionnaire completion or were participating in other psychological intervention studies.

Participants were recruited during routine IVF treatment appointments between March and December 2024. Consecutive sampling was used. All eligible women attending the clinics during the recruitment period were invited to participate. Recruitment materials, including brochures and posters, were displayed at the front nursing desk. The primary investigator supervised the recruitment process, while two trained research nurses briefly explained the study’s aims and procedures to interested women. Participants were asked to complete an online survey. They were informed that participation was voluntary, responses would remain confidential, and withdrawal could occur at any time without consequences for their ongoing treatment. Written informed consent was obtained from all participants before survey completion.

### 3.5. Measures

#### 3.5.1. Sociodemographic Information

A structured questionnaire was used to collect sociodemographic data, including age, education level, marital status, number of children, duration of infertility, and income satisfaction, and body mass index (BMI).

#### 3.5.2. Anxiety

Anxiety was measured using the Chinese version of the Zung Self-Rating Anxiety Scale (SAS) [20]. The SAS is a widely used self-report instrument designed to assess anxiety symptoms. It consists of 20 items rated on a 4-point Likert scale ranging from 1 = none or a little of the time to 4 = most or all of the time. Standard scores classified as follows: 50–59 = mild anxiety, 60–69 = moderate anxiety, and ≥70 = severe anxiety. Higher scores indicate greater levels of anxiety. The SAS has demonstrated acceptable reliability in infertile Chinese women [21]. In the present study, the Cronbach’s α was 0.724.

#### 3.5.3. Social Support

Social support was assessed using the Social Support Rating Scale (SSRS) [22]. The SSRS includes 10 items covering emotional, instrumental, and informational support, each rated on a 1–4 Likert scale. Total scores range from 10 to 40, with higher scores reflecting greater social support. The SSRS has been widely used in Chinese populations with strong psychometric properties. In the present study, the Cronbach’s α was 0.788.

#### 3.5.4. Family Resilience

Family resilience was measured using the Chinese version of the Family Resilience Assessment Scale (FRAS-C) [23]. The FRAS-C contains 32 items across three subscales: Family Communication and Problem Solving (FCPS), Utilizing Social Resources (USR), and Maintaining a Positive Outlook (MPO). Items are rated on a 4-point Likert scale (1 = strongly disagree to 4 = strongly agree). Total scores range from 32 to 128, with higher scores indicating greater family resilience. The FRAS-C has demonstrated satisfactory reliability and validity in Chinese populations. In the present study, the Cronbach’s α was 0.804.

#### 3.5.5. Quality of Life

Quality of life was measured using the Chinese version of the Fertility Quality of Life Questionnaire (FertiQoL) [24]. The FertiQoL consists of 36 items rated on a 5-point Likert scale (0 = not at all to 4 = very much). It includes six subscales (emotional, mind–body, relational, social, treatment environment, and treatment tolerability) and yields three summary scores (core FertiQoL, treatment FertiQoL, and total FertiQoL), all ranging from 0 to 100. Higher scores indicate better quality of life. The FertiQoL shows satisfactory reliability and validity in Chinese infertile populations [25]. In the present study, the Cronbach’s α was 0.811.

### 3.6. Data Analysis

All statistical analyses were conducted using R version 4.5.1 (R Core Team, 2025). The analytic workflow comprised descriptive statistics, correlation analyses, evaluation of multicollinearity and distributional assumptions, sequential mediation modeling, sensitivity analyses, and export of results for reporting. Missing data were handled using full information maximum likelihood, which uses all available information under the assumption that data are missing at random.

Continuous variables of primary interest included anxiety, social support, family resilience, and FertiQoL. Sociodemographic variables were recoded as numeric variables where appropriate for inclusion as covariates in regression and structural equation modeling (SEM) analyses. Categorical variables were dummy-coded for regression-based analyses and variance inflation factor (VIF) calculations.

Descriptive analyses were conducted for both sociodemographic and primary research variables. Means and standard deviations were calculated for continuous variables, whereas frequencies and percentages were reported for categorical variables. For the main study constructs, means, standard deviations, skewness, and kurtosis were calculated.

Pearson correlations were computed among the continuous study variables. Significance levels were estimated using bootstrap procedures where appropriate. Correlation matrices, including *p*-values, were exported for reporting.

VIFs were calculated for all predictors included in the SEM and sensitivity regression models, with VIF values greater than 5 considered indicative of potential multicollinearity [26]. Skewness and kurtosis were also examined for all continuous variables to assess distributional assumptions. Variables showing notable departures from normality were noted for interpretation and potential transformation.

A sequential mediation model was specified using the lavaan package. In this model, anxiety was treated as the independent variable, social support and family resilience as sequential mediators, and FertiQoL as the dependent variable. Covariates, including age, BMI, infertility duration, education, number of children, household income, and live births, were selected based on theoretical relevance and prior empirical evidence. Maximum likelihood estimation with robust standard errors was employed, and 5000 bootstrap resamples were used to generate bias-corrected confidence intervals for indirect effects. Standardized path coefficients (β) were reported. Model fit was evaluated using conventional indices, including the comparative fit index (CFI), Tucker–Lewis index (TLI), root mean square error of approximation (RMSEA), and standardized root mean square residual (SRMR). Specific mediation effects reported included: (a) the indirect effect via social support alone, (b) the indirect effect via the sequential pathway of social support → family resilience, (c) the total indirect effect, and (d) the total effect of anxiety on FertiQoL.

### 3.7. Sensitivity Analyses

Two sensitivity analyses were conducted to assess whether the mediation findings were robust to influential observations and alternative model specification. First, influential observations were identified using Cook’s distance from regression models predicting FertiQoL. Observations exceeding the conventional threshold of 4/n were excluded, and the mediation model was re-estimated. Second, an alternative mediator ordering was examined by specifying family resilience before perceived social support. This analysis was conducted to evaluate whether the results were sensitive to the assumed ordering of the mediators. Because the data were cross-sectional, these analyses were interpreted as exploratory model checks rather than tests of temporal or causal pathways.

## 4. Results

### 4.1. Descriptive Data

Of 425 eligible women approached, 419 consented and completed the online survey (response rate: 98.6%). The primary reasons for non-participation included a lack of time and a reluctance to join. Participants had a mean age of 33.30 years (SD = 4.66), a mean infertility duration of 3.42 years (SD = 2.89). Most participants had a college education (68%) and middle-income status (62%). Missing data ranged from 1.2% to 3.8% across variables and were handled using full information maximum likelihood in mediation analyses. See Table 1.

Anxiety scores ranged from 20 to 72 (M = 43.42, SD = 12.56). The majority scored in the mild anxiety range (50–59; N = 158, 37.7%). Moderate anxiety (60–69) was observed in 21 participants (5.0%), and severe anxiety (≥70) in 11 participants (2.6%).

### 4.2. Multicollinearity

VIFs were calculated for all predictors to assess multicollinearity. All VIFs were below the recommended cutoff of 5, indicating no significant multicollinearity concerns. The highest VIF was observed for social support (VIF = 3.03), followed by family resilience (VIF = 2.61) and anxiety (VIF = 2.07). All other variables, including age, BMI, infertility duration, education, number of children, number of live births, and household income, had VIFs between 1.02 and 1.46.

### 4.3. Distributional Assumptions

Skewness and kurtosis were examined for continuous variables to evaluate normality assumptions. Anxiety, social support, family resilience, and FertiQoL all demonstrated skewness values within ±1 and kurtosis values within ±2, suggesting acceptable univariate normality. Age also showed acceptable skewness (0.52) and kurtosis (0.24). However, BMI (skew = 2.05, kurtosis = 13.66) and infertility duration (skew = 2.23, kurtosis = 7.68) demonstrated significant positive skewness and leptokurtosis, indicating non-normal distributions. Given the robustness of bootstrapped SEM to violations of normality, these deviations were addressed through the use of bootstrapping procedures.

### 4.4. Correlation Analysis

Pearson correlations among the primary study variables are presented in Table 2. Anxiety was negatively correlated with social support (r = −0.70, *p* < 0.001), family resilience (r = −0.64, *p* < 0.001), and FertiQoL (r = −0.35, *p* < 0.001). Social support was positively correlated with family resilience (r = 0.77, *p* < 0.001) and with FertiQoL (r = 0.32, *p* < 0.001). Similarly, family resilience was positively associated with FertiQoL (r = 0.31, *p* < 0.001). These findings are consistent with the hypothesized relationships among study constructs. See Table 2.

### 4.5. Sequential Mediation Model

A sequential mediation model was tested to examine whether social support and family resilience mediated the relationship between anxiety and FertiQoL, controlling for age, BMI, infertility duration, education, number of children, number of live births, and household income. The model was estimated using maximum likelihood with 5000 bootstrap samples, and missing data were handled via full information maximum likelihood (FIML). See Table 3.

#### 4.5.1. Model Fit

The hypothesized sequential mediation model provided an adequate fit to the data: χ^2^(26) = 34.21, *p* = 0.12, CFI = 0.97, TLI = 0.95, RMSEA = 0.028, 90% CI [0.000, 0.056], SRMR = 0.034. These indices indicate a good overall model fit according to conventional cutoffs (CFI and TLI ≥ 0.95, RMSEA ≤ 0.06, SRMR ≤ 0.08). Given the acceptable fit, subsequent path coefficients were interpreted for mediation effects.

#### 4.5.2. Direct Associations

Anxiety was negatively associated with social support, B = −0.62, SE = 0.03, *p* < 0.001, standardized β = −0.68, indicating that higher anxiety was associated with lower social support. Social support was positively associated with family resilience, B = 1.95, SE = 0.14, *p* < 0.001, β = 0.63, while anxiety was negatively associated with family resilience, B = −0.51, SE = 0.13, *p* < 0.001, β = −0.18. Anxiety also showed a negative association with FertiQoL, B = −0.37, SE = 0.09, *p* < 0.001, β = −0.23. The direct associations of social support (B = 0.14, SE = 0.12, *p* = 0.26, β = 0.08) and family resilience (B = 0.06, SE = 0.04, *p* = 0.17, β = 0.09) with FertiQoL were not statistically significant.

#### 4.5.3. Indirect and Total Associations

Bootstrapped analyses indicated the following:

Anxiety → Social support → FertiQoL: B = −0.08, SE = 0.08, 95% CI [−0.23, 0.06], *p* = 0.27

Anxiety → Social support → Family resilience → FertiQoL: B = −0.07, SE = 0.05, 95% CI [−0.18, 0.03], *p* = 0.17

Neither individual indirect path was statistically significant, indicating that the sequential mediation model was not supported.

Total indirect association: B = −0.15, SE = 0.07, 95% CI [−0.28, −0.01], *p* = 0.03. This suggests an overall indirect association involving anxiety, social support, family resilience, and FertiQoL, although the specific stepwise pathway through social support and family resilience was not confirmed.

**Figure 2:**
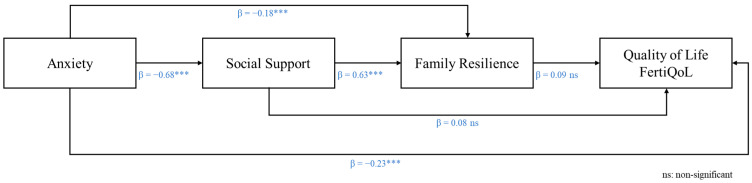
Results of the Sequential Mediation Model. *** *p* ≤ 0.001.

The total association between anxiety and FertiQoL was significant, B = −0.52, SE = 0.06, *p* < 0.001, β = −0.32, indicating that higher anxiety was associated with lower FertiQoL overall. See Figure 2.

#### 4.5.4. Covariates

Among covariates, higher education levels predicted greater social support (B = 0.94, SE = 0.38, *p* = 0.01, β = 0.10). Number of children positively predicted FertiQoL (B = 4.24, SE = 2.08, *p* = 0.04, β = 0.10), whereas higher income satisfaction negatively predicted FertiQoL (B = −3.47, SE = 1.56, *p* = 0.03, β = −0.11). Other covariates were non-significant.

### 4.6. Results of Sensitivity Analysis

Two sensitivity analyses were conducted to examine the robustness of the mediation findings.

First, influential observations were identified using Cook’s distance from regression models predicting FertiQoL. Observations exceeding the conventional threshold of 4/n were excluded, and the mediation model was re-estimated. The direction of the key paths was generally consistent with the main analysis. Anxiety remained negatively associated with social support (β = −0.63, *p* < 0.001), and social support remained positively associated with family resilience. The path from family resilience to FertiQoL remained positive but was not statistically significant (β = 0.09, *p* = 0.17). Unlike the primary analysis, the sequential indirect effect of anxiety on FertiQoL through social support and family resilience reached statistical significance after influential observations were removed. This finding suggests that the sequential indirect effect may be sensitive to influential observations and should therefore be interpreted cautiously.

Second, an alternative mediator ordering was examined by specifying family resilience before social support. In this model, anxiety was negatively associated with family resilience (β = −0.64, *p* < 0.001) and social support (β = −0.35, *p* < 0.001), and family resilience was positively associated with social support (β = 0.55, *p* < 0.001). Anxiety remained negatively associated with FertiQoL (β = −0.23, *p* < 0.001). However, the direct paths from family resilience to FertiQoL (β = 0.11, *p* = 0.10) and from social support to FertiQoL (β = 0.08, *p* = 0.25) were not statistically significant. The sequential indirect effect of anxiety on FertiQoL through family resilience and social support was also not statistically significant (β = −0.03, *p* = 0.25, 95% CI [−0.12, 0.03]). See Table 4.

Overall, the sensitivity analyses did not provide consistent support for a stable sequential mediation pathway. Although several path directions were consistent with the theoretical model, the sequential indirect effect was not robust across model specifications.

## 5. Discussion

This study examined associations among anxiety, social support, family resilience, and FertiQoL in women undergoing IVF. A theory-guided mediation model was explored to determine whether social support and family resilience were involved in indirect associations between anxiety and FertiQoL.

### 5.1. Anxiety

In the present study, most participants exhibited mild anxiety, while moderate and severe anxiety were relatively uncommon. This distribution suggests that although infertility imposes psychological stress, the majority of women experienced anxiety at a manageable level. The concentration of scores in the mild range is consistent with prior research indicating that women undergoing fertility treatment often report heightened but generally moderate anxiety levels [27]. Nevertheless, the presence of moderate and severe anxiety in a subset of participants highlights the need for targeted psychosocial support, particularly for those at higher risk of psychological distress.

### 5.2. Direct Associations

Consistent with prior research, anxiety was negatively associated with quality of life, indicating that higher anxiety corresponds to lower perceived well-being during IVF treatment [28]. This finding is clinically relevant because IVF is often accompanied by multiple stresses [29], such as emotional uncertainty. In this context, anxiety may reflect not only psychological distress but also broader difficulties in coping with infertility-related stress. The finding indicates that anxiety and poorer FertiQoL co-occur in this population, supporting the importance of assessing psychological distress during IVF care [30].

Although social support and family resilience were significantly correlated with FertiQoL in the bivariate analyses, their direct paths to FertiQoL were not statistically significant in the adjusted sequential mediation model. This discrepancy is likely because Pearson correlations reflect unadjusted pairwise associations, whereas the mediation model estimates the unique association of each predictor with FertiQoL while accounting for anxiety, the other mediator, and covariates. The nonsignificant direct paths therefore suggest that the associations of social support and family resilience with FertiQoL may be partly attributable to shared variance with anxiety or conceptual overlap between the two psychosocial resource variables. These findings indicate that social support and family resilience may be relevant to FertiQoL, but their independent roles as proximal predictors were not supported in the adjusted model.

### 5.3. Psychosocial Mechanisms: Social Support and Family Resilience

While the hypothesized sequential pathway (Anxiety → Social support → Family resilience → FertiQoL) was not statistically supported, the total indirect effect was significant. This finding suggests the presence of an overall indirect association involving anxiety, social support, family resilience, and FertiQoL. However, because the specific indirect effects were not statistically significant, the results do not provide clear evidence that social support and family resilience operate as sequential mediators in the proposed order.

Several factors may explain the lack of support for the stepwise mediation. First, the cross-sectional design limits the ability to capture temporal dynamics, which may be essential for sequential processes; the development of family resilience in response to social support may occur over time rather than instantaneously, making it difficult to detect in a single time-point assessment. Second, measurement limitations could have reduced sensitivity to subtle associations; for example, self-report instruments may be influenced by reporting biases or may not fully capture the complexity of family resilience as it evolves in response to stress. Third, unmeasured variables, such as partner support, coping strategies, or cultural factors, may influence the relationships between anxiety, social support, and family resilience, potentially obscuring the sequential pathway. Finally, the strength of individual effects may vary across participants, and the sequential effect may be smaller than detectable in this sample size.

Social support theory posits that support from networks, including emotional, informational, and instrumental support, enhances coping and reduces distress [31]. The negative association between anxiety and social support suggests that women with higher anxiety reported lower perceived availability or adequacy of social resources. Family resilience theory emphasizes adaptive family functioning emerging from internal strengths and external resources, enabling families to collectively manage stress [32]. The positive association between social support and family resilience suggests that individual-level perceived resources may be linked to broader family-level adaptive capacity.

Nevertheless, the nonsignificant direct paths from social support and family resilience to FertiQoL indicate that these constructs did not function as strong predictors of FertiQoL in the adjusted model. Therefore, the findings should not be interpreted as confirming a sequential mediation mechanism. Instead, they suggest that anxiety, social support, and family resilience are interrelated psychosocial factors that may be relevant to women’s FertiQoL. Future longitudinal studies are needed to determine whether these factors operate sequentially over time and whether changes in social support or family resilience are followed by changes in FertiQoL.

### 5.4. Covariates and Socioeconomic Factors

The analysis of covariates revealed several nuanced predictors of psychosocial outcomes. The finding that higher education predicted greater social support aligns with existing literature, suggesting that educational attainment may equip individuals with stronger social networks or more advanced communication skills to seek and secure support during stressful life events like infertility treatment [33]. Interestingly, while having more children was associated with a higher quality of life specific to FertiQoL, potentially providing a sense of existing familial fulfillment that buffers against the distress of infertility, higher income satisfaction unexpectedly predicted a lower FertiQoL. This counterintuitive result may be explained by the “paradox of privilege”; While many studies report that lower incomes are associated with greater psychological distress among infertile individuals [10], it is possible that among those with greater financial resources, distress may also be elevated, potentially driven by higher expectations of success or a sense of injustice when infertility persists despite available means. These findings underscore the complex and sometimes non-linear relationship between socioeconomic factors and psychological adaptation to infertility.

### 5.5. Sensitivity Analyses and Alternative Sequential Ordering

The sensitivity analyses further support a cautious interpretation of the mediation findings. Although the sequential indirect effect reached statistical significance after influential observations were removed, this differed from the primary analysis, in which the specific sequential indirect effect was not significant. Moreover, the alternative mediator-ordering model did not show a significant sequential indirect effect. These findings suggest that the proposed sequential pathway may be sensitive to model specification and influential observations. Longitudinal studies are needed before concluding mediator ordering or temporal pathways.

### 5.6. Implications

The findings of this study suggest that assessment of anxiety, social support, and family resilience may help identify women who have greater psychosocial needs during IVF. Supportive strategies such as counseling, peer support, and partner or family involvement may be considered as part of holistic infertility care. However, given the cross-sectional design and the nonsignificant specific indirect pathways, these findings should not be interpreted as evidence that enhancing social support or family resilience will directly improve FertiQoL. Longitudinal and intervention studies are needed to determine whether these psychosocial resources are modifiable factors that may be associated with better outcomes over time.

Theoretically, the findings highlight the value of examining FertiQoL within a broader psychosocial context that includes individual distress, interpersonal support, and family adaptive capacity. Also, the lack of support for the proposed sequential pathway suggests that the relationships among these constructs may be more complex, reciprocal, or context-dependent than initially hypothesized. Future research should examine alternative model structures, subscale-level associations, longitudinal pathways, and the role of cultural, relational, and treatment-related factors in shaping social support and family resilience during fertility treatment.

### 5.7. Limitations

Several limitations should be noted. First, the cross-sectional design precludes causal inference. Although a sequential mediation model was tested, the results should be interpreted as theoretically informed associations rather than confirmed causal pathways. Temporal ordering among anxiety, social support, family resilience, and QoL cannot be established, and longitudinal or experimental studies are needed to validate the hypothesized directionality. Second, while demographic and treatment-related covariates were included, potential moderators, such as prior fertility treatments, partner characteristics, or additional psychosocial factors, may influence these associations and should be investigated in larger, more diverse samples. Last, this study focused on total scale scores rather than subscale-level analyses. Future research could incorporate subscale variables, as well as additional psychological and contextual constructs, such as coping strategies, partner support, and cultural beliefs, to develop a more comprehensive understanding of resilience in fertility treatment contexts.

## 6. Conclusions

In summary, this study found that anxiety was negatively associated with FertiQoL among women undergoing IVF. Anxiety was also associated with lower social support and family resilience. Although the total indirect association involving social support and family resilience was statistically significant, the hypothesized sequential mediation pathway was not supported by the specific indirect effects. Therefore, the findings should be interpreted as evidence of interrelated psychosocial factors rather than confirmation of a sequential mediation mechanism.

These results may help inform future research on psychosocial adjustment in infertility care by identifying anxiety, social support, and family resilience as relevant domains for further investigation. Longitudinal and intervention studies are needed to clarify the temporal ordering, causal mechanisms, and clinical significance of these relationships.

## Figures and Tables

**Table 1:** Social-Demographic Characteristics of Study Participants (N = 419).

Variable	Category	Count	Percentage	Mean ± SD/Range
Age				33.30 ± 4.66 years (20–49)
Infertility History				3.42 ± 2.89 years
Education	Below primary	49	11.69%	
	High school	42	10.02%	
	College	99	23.63%	
	Bachelor	161	38.42%	
	Postgraduate	68	16.23%	
Number of Children	0	358	85.44%	
	1	46	10.98%	
	2	14	3.34%	
	More than 2	1	0.24%	
Marital Status	Married	367	87.59%	
	Remarried	52	12.41%	
Living Arrangement	Urban	352	84.01%	
	Rural	67	15.99%	
Ability of Income to Support Treatment	Fully sufficient	79	18.85%	
	Sufficient	302	72.08%	
	Somewhat difficult	17	4.06%	
	Very difficult	21	5.01%	
Anxiety				43.42 ± 12.56
Social support				25.91 ± 11.31
Family resilience				81.63 ± 35.06
FertiQoL				61.98 ± 20.29

**Table 2:** Correlations Among Primary Study Variables.

Variable	1	2	3	4
1. Anxiety	—			
2. Social support	−0.70 ***	—		
3. Family resilience	−0.64 ***	0.77 ***	—	
4. FertiQoL	−0.35 ***	0.32 ***	0.31 ***	—

*** *p* ≤ 0.001.

**Table 3:** Sequential Mediation Model Parameter Estimates (Standardized Coefficients).

Outcome	Predictor	β	*p*
Social support	Anxiety	−0.68	<0.001
Social support	Education	0.10	0.014
Family resilience	Social support	0.63	<0.001
Family resilience	Anxiety	−0.18	<0.001
FertiQoL	Anxiety	−0.23	<0.001
FertiQoL	Social support	0.08	0.262
FertiQoL	Family resilience	0.09	0.170
Indirect	Anxiety → Social support → FertiQoL	−0.05	0.268
Indirect	Anxiety → Social support → Family resilience → FertiQoL	−0.04	0.173
Total indirect	Anxiety → FertiQoL	−0.09	0.026
Total effect	Anxiety → FertiQoL	−0.32	<0.001

Note. β = standardized coefficient. N = 419. Indirect effects estimated via 5000 bootstrap samples. Covariates (age, BMI, infertility duration, number of children, live births, household income) are included in the model but omitted here for clarity.

**Table 4:** Standardized and Unstandardized Estimates for the Alternative Sequential Mediation Model.

Outcome	Predictor	B	SE	z	*p*	95% CI (Lower, Upper)	Std. β
Family Resilience	Anxiety	−1.77	0.10	−17.11	<0.001	−1.98, −1.57	−0.64
Social Support	Family Resilience	0.18	0.02	10.22	<0.001	0.14, 0.21	0.55
Social Support	Anxiety	−0.32	0.05	−6.33	<0.001	−0.41, −0.21	−0.35
FertiQoL	Anxiety	−0.37	0.09	−3.95	<0.001	−0.55, −0.18	−0.23
FertiQoL	Family Resilience	0.06	0.04	1.64	0.101	−0.01, 0.14	0.11
FertiQoL	Social Support	0.14	0.12	1.16	0.246	−0.10, 0.36	0.08
Indirect effect	Anxiety → Family Resilience → Social Support → FertiQoL	−0.04	0.04	−1.15	0.250	−0.12, 0.03	−0.03

## Data Availability

The data presented in this study are available on request from the corresponding author due to ethical reasons.

## References

[1] World Health Organization (2023). Infertility Prevalence Estimates, 1990–2021.

[2] Zhu C., Yan L., He C., Wang Y., Wu J., Chen L., Zhang J. (2022). Incidence and risk factors of infertility among couples who desire a first and second child in Shanghai, China: A facility-based prospective cohort study. Reprod. Health.

[3] Bindeman J., Abbasi R., Sacks P.C. (2025). The Mental Health Traumas of Infertility: Impact and Consequences. Obstet. Gynecol. Clin. N. Am..

[4] Paraskevi L., Antigoni S., Kleanthi G. (2021). Stress and Anxiety Levels in Couples who Undergo Fertility Treatment: A Review of Systematic Reviews. Mater. Socio Medica.

[5] Logan S., Gu R., Li W., Xiao S., Anazodo A. (2019). Infertility in China: Culture, society and a need for fertility counselling. Asian Pac. J. Reprod..

[6] Wang L., Tang Y., Wang Y. (2023). Predictors and incidence of depression and anxiety in women undergoing infertility treatment: A cross-sectional study. PLoS ONE.

[7] Chaurasiya S., Singh R., Bahadur B., Singh S., Maurya V., Rai S. (2025). Psychological distress in women with primary and secondary infertility: A comparative analysis of depression, anxiety, and stress. Front. Public Health.

[8] Zurlo M.C., Cattaneo Della Volta M.F., Vallone F. (2020). Infertility-Related Stress and Psychological Health Outcomes in Infertile Couples Undergoing Medical Treatments: Testing a Multi-dimensional Model. J. Clin. Psychol. Med. Settings.

[9] Braverman A.M., Davoudian T., Levin I.K., Bocage A., Wodoslawsky S. (2024). Depression, anxiety, quality of life, and infertility: A global lens on the last decade of research. Fertil. Steril..

[10] Bagade T., Mersha A.G., Majeed T. (2023). The social determinants of mental health disorders among women with infertility: A systematic review. BMC Women’s Health.

[11] Kort-Butler L.A. (2017). Social Support Theory. The Encyclopedia of Juvenile Delinquency and Justice.

[12] Lopez R.B., Courtney A.L., Liang D., Swinchoski A., Goodson P., Denny B.T. (2024). Social support and adaptive emotion regulation: Links between social network measures, emotion regulation strategy use, and health. Emotion.

[13] Ghasemi B., Riazi H., Alamolhoda S.H., Montazeri A. (2025). Social Support, Mental Health, and Quality of Life in Women Experiencing Infertility: A Systematic Review. Health Soc. Care Community.

[14] Henry C.S., Harrist A.W., Adamsons K., Few-Demo A.L., Proulx C., Roy K. (2022). Family Resilience Theory. Sourcebook of Family Theories and Methodologies: A Dynamic Approach.

[15] Lam B.-H., Lam B.-H. (2019). Well-being, Psychological Adjustments and Effective Social Support Giving. Social Support, Well-Being, and Teacher Development.

[16] Casaburo G., Yzaguirre M., Subramaniam S., Holtrop K. (2023). A Systematic Review of Family Stress Theory in Mental Health Research (2010–2020). Fam. Soc. J. Contemp. Soc. Serv..

[17] Peleg O., Peleg M. (2025). Is Resilience the Bridge Connecting Social and Family Factors to Mental Well-Being and Life Satisfaction?. Contemp. Fam. Ther..

[18] Kang X., Fang M., Li G., Huang Y., Li Y., Li P., Wang H. (2022). Family resilience is a protective buffer in the relationship between infertility-related stress and psychological distress among females preparing for their first in vitro fertilization-embryo transfer. Psychol. Health Med..

[19] Sim M., Kim S.Y., Suh Y. (2022). Sample Size Requirements for Simple and Complex Mediation Models. Educ. Psychol. Meas..

[20] Zung W.W. (1971). A rating instrument for anxiety disorders. Psychosomatics.

[21] Xu H., Ouyang N., Li R., Tuo P., Mai M., Wang W. (2017). The effects of anxiety and depression on in vitro fertilisation outcomes of infertile Chinese women. Psychol. Health Med..

[22] Xiao S. (1994). Theoretical basis and research application of social support rating scale. J. Clin. Psychiatry.

[23] Li Y., Zhao Y., Zhang J., Lou F., Cao F. (2016). Psychometric Properties of the Shortened Chinese Version of the Family Resilience Assessment Scale. J. Child Fam. Stud..

[24] Boivin J., Takefman J., Braverman A. (2011). The fertility quality of life (FertiQoL) tool: Development and general psychometric properties. Hum. Reprod..

[25] Bai J., Zheng J., Guo N., Dong Y., Wang K., Cheng C., Jiang H., Qian L. (2024). Coping Profiles and Differences in Psychological Distress and Quality of Life in Clients Undergoing Assisted Reproductive Techniques: A Latent Profile Analysis. J. Multidiscip. Healthc..

[26] Kim J.H. (2019). Multicollinearity and misleading statistical results. Korean J. Anesthesiol..

[27] Gui W., Yang X., Jiang H., Wu H., Zeng M., Wen Y., Qiu T., Zhang Y., Ma Z., Tong C. (2021). Prevalence of anxiety and its associated factors among infertile patients after ‘two-child’ policy in Chongqing, China: A cross-sectional study. Reprod. Health.

[28] Ye H., Zhao J., Zou Y., Song X., Xu M., Zhang Y., Zhang L., Wang G. (2025). Correlations among hope, fertility quality of life and negative emotions for couples undergoing their first in vitro fertilization–embryo transfer: A cross-sectional analysis. Reprod. Health.

[29] Jin R., Wang P., Shan Y., Lai W. (2025). Current psychological symptoms and emotional distress in women undergoing IVF-ET: An investigation and correlation analysis of medical care, family care and social support. Cure Care.

[30] Holley S.R., Pasch L.A., Domar A.D. (2021). Dealing with the emotional distress following failed IVF. Assisted Reproduction Techniques.

[31] Acoba E.F. (2024). Social support and mental health: The mediating role of perceived stress. Front. Psychol..

[32] Walsh F. (2021). Family resilience: A dynamic systemic framework. Multisystemic Resilience: Adaptation and Transformation in Contexts of Change.

[33] Hahn R.A., Truman B.I. (2015). Education Improves Public Health and Promotes Health Equity. Int. J. Health Serv..

